# BMC Complementary and Alternative Medicine Reviewer Acknowledgement, 2013

**DOI:** 10.1186/1472-6882-14-13

**Published:** 2014-01-20

**Authors:** Thomas A Rowles

**Affiliations:** 1 BioMed Central, Floor 6 236 Gray’s Inn Road, London, WC1X 8HB, United Kingdom

## Abstract

**Contributing reviewers:**

The editors of BMC Complementary and Alternative Medicine would like to thank all our reviewers who have contributed their time to the journal in Volume 13 (2013).

## 

Omar Abdel Salam

Egypt

Azza Abdel-Aty

Egypt

Nahla Abdel-Azim

Egypt

Shereen Abdelghaffar

Egypt

Maged Abdel-Kader

Saudi Arabia

Ihab Abdel-Raheem

Egypt

Mahmood Abdulla

Malaysia

Anuruddha Mahasen Abeygunasekera

Sri Lanka

Nisen Abuaf

France

Sawsan Abuhamdah

Jordan

Abuduaini Abulimiti

USA

Dario Acuña-Castroviejo

Spain

Ernest Adeghate

United Arab Emirates

Adejuwon Adeneye

Nigeria

Sunday Adesegun

Nigeria

Adewalw Adetutu

Nigeria

Stephen Adewole

Nigeria

Emmanuel Adewusi

South Africa

Farrukh Afaq

USA

Milee Agarwal

India

Ramesh Chandra Agrawal

India

Paban Agrawala

India

Joseph K Agyin

USA

Aamir Ahmad

USA

Bashir Ahmad

Pakistan

Dilshad Ahmad

Saudi Arabia

Augustine Ahmadu

Nigeria

Hanaa Ahmed

Egypt

Mikel Aickin

USA

Alfredo Aires

Portugal

Haji Akber Aisa

China

Olayinka Aiyegoro

South Africa

Gülþen Akalýn

Turkey

Dickens Akena

Uganda

Hulya Aksoy

Turkey

Fouad Al- Bayaty

Malaysia

A.H.M.K. Alam

Bangladesh

Samir Aleryani

USA

Angel Alex

India

Matthew Alexander

USA

Abbeer Alhadi

Malaysia

Mahmoud Alhosin

France

Mamdouh Ali

Egypt

Muhammad Ali

Pakistan

Niaz Ali

Pakistan

Malona Alinsug

Taiwan

Mohammed Ali-Shtayeh

Palestinian Territory

Romulo Alves

Brazil

Narasimhacharya Amaravadi V.R.L.

India

Ryszard Amarowicz

Poland

Mohan Amberkar

India

Omar Ameer

Australia

Saeed Amel Jamedar

Iran

Steven Ames

USA

Arm Amin

USA

Nacoulma Aminata

Belgium

Norhaniza Aminudin

Malaysia

Stephen Amoo

South Africa

Hyojin An

South Korea

Arturo Anadon

Spain

Madhu Anand-Srivastava

Canada

M. Robyn Andersen

USA

Richard Anderson

USA

Emmanouil Apostolidis

USA

Chippada Appo

India

Sandra Arandelovic

Serbia

Jesil Aranjani

India

Carlos Areche

Chile

Adeyemi Oladapo Aremu

South Africa

Ebru Arioglu Inan

Turkey

Andrea Aro

Brazil

Rajesh Arora

India

Mavis Asare

Ghana

Vasumathi Asha

India

Tom Ashafa

South Africa

Gary Asher

USA

Tulin Askun

Turkey

Areej Assaf

Jordan

Jules Clement Nguedia Assob

Cameroon

Akram Astani

Iran

Humberto Astiazaran-Garcia

Mexico

Athar Ata

Canada

Forough Ataollahi

Malaysia

Simon Kwaku Attah

Ghana

Ingvild Austarheim

Norway

Frederick Ausubel

USA

Celestine Chukwuemeke Azikiwe

Nigeria

Koichi Baba

Japan

Jegdish Babu

USA

Horacio Bach

Canada

Yong Hyeon Baek

South Korea

Enitome Bafor

Nigeria

Vivek K. Bajpai

South Korea

Madhu Bala

India

R. Balaraman

India

Manjeshwar Shrinath Baliga

India

Lynda Balneaves

Canada

Kirnpal-Kaur Banga Singh

Malaysia

Sarang Bani

India

Luís Barbisan

Brazil

Letricia Barbosa-Pereira

Spain

Luciola Barcelos

Brazil

Tatiana Barichello

Brazil

Jo Barnes

New Zealand

Ines Barone

Italy

Maria Do Carmo Barreto

Portugal

Ronald Bartels

Netherlands

K. Husnu Can Baser

Turkey

Samra Bashir

Pakistan

Ronan Batista

Brazil

Maurizio Battino

Italy

Michael Bauer

Australia

Saw Bawm

Myanmar

David Becker

USA

Luis Miguel Bedoya

Spain

Iris Bell

USA

Yuva Bellik

Algeria

Obdulio Benavente-García

Spain

Sandor Beniczky

Denmark

Kirsten Benkendorff

Australia

Vance Berger

USA

Jurga Bernat

Lithuania

Andresa Berretta

Brazil

David Betancur-Ancona

Mexico

Daniel Bezerra

Brazil

Ira Bhatnagar

India

Piyali Bhattacharyya

Puerto Rico

Joel Bialosky

USA

Zhaoxiang Bian

Hong Kong

Stephen Birch

Netherlands

Oddveig Birkeflet

Norway

Prakash Bisen

India

Felicity Bishop

United Kingdom

Subrata Kumar Biswas

Bangladesh

William Blaner

USA

Liu Bo

China

Virgilio Bocanegra-Garcia

Mexico

Ebru Bodur

Turkey

Katja Boehm

Germany

Bradley Bolling

USA

Leoni Bonamin

Brazil

Heather Boon

Canada

Patcharee Boonsiri

Thailand

Eric Borsting

USA

Mohammad Hossein Boskabady

Iran

Ryan Bradley

USA

Alex Braiman

Israel

Lesley Braun

Australia

Caragh Brosnan

Australia

Lindsay Brown

Australia

Luigi Brunetti

Italy

Diego Bucci

Italy

Hudson Buck

Brazil

Arndt Buessing

Germany

Armando Burgos-Hernandez

Mexico

Célia Cabral

Portugal

Daniela Cabrini

Brazil

Elif Cadirci

Turkey

Gioacchino Calapai

Italy

Marcus Calkins

USA

Jose Camara

Portugal

David Cameron-Smith

New Zealand

Chunlei Cang

USA

Antonella Canini

Italy

Edgar Cano-Europa

Mexico

Huijuan Cao

China

Peng Cao

China

Wangsen Cao

China

Wenqing Cao

USA

Marcia Carneiro

Brazil

Christine Carson

Australia

Brenda Cartmel

USA

Tina Cartwright

United Kingdom

Marcelo Carvalho

Brazil

Adelaida María Castro-Sánchez

Spain

Mauro Ceccanti

Italy

Roberto Cedillo-Rivera

Mexico

Younbyoung Chae

South Korea

Olga Chaim

Brazil

Raja Chakraborty

India

Wei Chiang Chan

Malaysia

Shun-Wan Chan

China

Sue Hay Chan

Malaysia

Chanpen Chanchao

Thailand

Dhyan Chandra

USA

Fang-Rong Chang

Taiwan

Hsiao-Yun Chang

Taiwan

Jia-Ming Chang

Taiwan

Ki Churl Chang

South Korea

Lee-Tian Chang

Taiwan

Shih-Liang Chang

Taiwan

Sungwon Chang

Australia

Yuan Shiun Chang

Taiwan

Ling Changquan

China

Jane Chao

Taiwan

Li-Fen Chao

Taiwan

Maria Chao

USA

Sreemoyee Chaterjee

India

Arnaud Chatonnet

France

Pronobesh Chattopadhyay

India

Porntip Chavalitshewinkoon-Petmitr

Thailand

Chun-Tao Che

USA

Chao-Jung Chen

Taiwan

Ching-Hsein Chen

Taiwan

Chinhui Chen

Taiwan

Chung-Yi Chen

Taiwan

Fang-Pey Chen

Taiwan

Guoxun Chen

USA

Jaw-Wen Chen

Taiwan

Jiachun Chen

China

Jia-Xu Chen

China

Julie Chen

Hong Kong

Kevin Chen

USA

Lixin Chen

China

Qingyong Chen

China

Shao-Nong Chen

USA

Taoyong Chen

China

Tzeng-Ji Chen

Taiwan

Vincent Chen

USA

Wei Chen

China

Weiqing Chen

China

Yann-Jang Chen

Taiwan

Yi-Hung Chen

Taiwan

Hao Yuan Cheng

Taiwan

Juei-Tang Cheng

Taiwan

Ling Cheng

USA

Steven Cheng

USA

Dan Cherkin

USA

Corjena Cheung

USA

Adeline Chia

Malaysia

Chih-Yen Chien

Taiwan

Mohankumar Chinnamma

India

Mallikarjun Chitneni

Malaysia

Hye-Youn Cho

USA

Jae Youl Cho

South Korea

Somi Cho

South Korea

Inhwa Choi

South Korea

Sun-Mi Choi

South Korea

Yung Hyun Choi

South Korea

Pei Pei Chong

Malaysia

Vui Heng Chong

Brunei

Fen-Pi Chou

Taiwan

Jude Chukwujekwu

South Africa

Kwang Chul Chung

South Korea

Vincent Chung

Hong Kong

Sasitorn Chusri

Thailand

Richard Cimanga Kanyanga

Belgium

Yuri Clement

Trinidad and Tobago

Ahmet Yilmaz Coban

Turkey

Sue Cochrane

Australia

Debbie Cohen

USA

Preciosa Coloma

Netherlands

Angel Concheiro

Spain

Kieran Cooley

Canada

Werner Cordier

South Africa

Leah Cosgrove

Australia

Samantha Coulson

Australia

Maria Alice Cruz-Hofling

Brazil

Hannah Dahlen

Australia

Haibin Dai

China

Zhijun Dai

China

Marco Dalla-Rizza

Uruguay

Regina Day

USA

Jillian De Gezelle

USA

Laura De Martino

Italy

Alberto De Oliveira

Brazil

Carolina De Oliveira

Brazil

Giuseppina De Petro

Italy

Arnaud Dechamps

Indonesia

Gagan Deep

USA

Felipe De-Faria

Brazil

Mariam Degani

India

Kayena Delaix Zaqueo

Brazil

Afef Dellai

Yemen

Jou-Fang Deng

Taiwan

Xuming Deng

China

Krupali Desai

USA

Preeti Desai

India

Miranda Deutschlander

South Africa

Saikat Dewanjee

India

Annette Dickinson

USA

Jacqueline Dimatelis

South Africa

Maurizio D'Incalci

Italy

Pei-Hui Ding

China

Ralf Dittrich

Germany

Anuja Dokras

USA

Fernanda Domingues

Portugal

Guangzhi Dong

South Korea

Tara Donker

Australia

Anton Dormer

USA

Guan-Hua Du

China

Jin Ao Duan

China

Vikash Kumar Dubey

India

Jean-Jacques Dugoua

Canada

Pin-Der Duh

Taiwan

Radhika Durai

India

Veeramuthu Duraipandiyan

India

Belma Durupinar

Turkey

Jeffery Dusek

USA

Som Dutt

India

Nidhi Dwivedi

India

Ana Dzamic

Serbia

Jean Paul Dzoyem

Cameroon

Mohammad Ali Ebrahimzadeh

Iran

Tamer Edirne

Turkey

Melike Ekizoglu

Turkey

M A El Missiry

Egypt

Muhammad El Shafeey Ahmed El Shafeey

Egypt

Selvakumar Elangovan

India

Mohamed El-Boshy

Egypt

William Elder

USA

Kawther El-Gendy

Egypt

Anat Elmann

Israel

Sahar Elmekkawy

Egypt

Reem El-Naga

Egypt

Wael El-Tras

Egypt

Ephrem Engidawork

Ethiopia

Hui Meng Er

Malaysia

Esra Eroglu Ozkan

Turkey

Özlem Erol

Turkey

Pilar Escolar-Reina

Spain

Javier Espinosa-Aguirre

Mexico

Manuel Estrada

Chile

Sue Evans

Australia

Fabio Facchinetti

Italy

Enrico Facco

Italy

Mehran Fadaeinasab

Malaysia

Titilayo Fakeye

Nigeria

Hanen Falleh

Tunisia

Bo Guang Fan

Canada

Xiaohui Fan

China

Yihui Fan

USA

Yi-Ming Fan

China

Evandro Fang

USA

Samir Farghaly

USA

Júlia Farias

Brazil

Tahereh Farkhondeh

Iran

Elizaveta Fasler-Kan

Switzerland

Olaniyi Fawole

South Africa

Bibi Sedigheh Fazly Bazzaz

Iran

Jia Fei

China

Yutong Fei

China

Yibin Feng

Hong Kong

Patricia Fernandes

Brazil

Matilde Fernandez

Spain

Andrea Ferreira

Brazil

Maria Pontes Ferreira

USA

Raina Fichorova

USA

Florêncio Figueiredo

Brazil

Regina Figueiredo

Brazil

Vittorio Fineschi

Italy

Peter Fisher

United Kingdom

Margaret Fitch

Canada

Tatiana Fiuza

Brazil

Nicholas Fleming

New Zealand

Andrew Flower

United Kingdom

Mary Foglio

Brazil

Anelise Formagio

Brazil

Moshe Frenkel

USA

Ulrich Frey

Germany

Robert Friedman

USA

Jeff Fry

United Kingdom

Dietmar Fuchs

Austria

Hirotaka Fukasawa

Japan

Hiroyuki Fukui

Japan

Ricardo Furtado

Brazil

Weidao Ga

India

Goran Gajski

Croatia

Hala Gali-Muhtasib

Lebanon

Luca Gallelli

Italy

Zhan-Guo Gao

USA

Gabriela Garcia

USA

M. Kay Garcia

USA

Virginia Garcia

Spain

Paula Gardiner

USA

Pietro Gareri

Italy

Lilach Gavish

Israel

Yasin Genç

Turkey

Maureen George

USA

Max Geraedts

Germany

Lia Noemi Gerschenson

Argentina

Jeffrey Gertsch

USA

Jawhar Gharbi

Tunisia

Irfan Ahmad Ghazi

India

Gabriele Ghisleni

Brazil

Jean Giacomotto

Australia

Nicola Giordano

Italy

Goezde Girgin

Turkey

Erika Gisel

Canada

Viswanatha Gl

India

Raj Goel

India

Alfred Goldberg

USA

John Golding

United Kingdom

Cheng-Xin Gong

USA

Margarete Goppelt-Struebe

Germany

Hiroaki Goto

Japan

Kazuo Goto

Japan

Suzanne Grant

Australia

Caroline Gray

USA

Carol Greco

USA

Sheila Greenfield

United Kingdom

Jeffrey Greeson

USA

Daniel Grenier

Canada

Erik Groessl

USA

Maria Groot

Netherlands

Amelia Guadalupe-Grau

Denmark

Corina Guethlin

Germany

Gunjan Guha

USA

César Luiz Guimarães

Brazil

Lamia Guizani-Tabbane

Tunisia

Mir Zahoor Gul

India

Ilhami Gulcin

Turkey

Muhammad Gulfraz

Pakistan

Zhi-Ling Guo

USA

Digant Gupta

USA

Praveena Gupta

USA

Sanjay Gupta

USA

Esther Guzman

USA

Hong-Seok Ha

South Korea

Guy Haegeman

Belgium

Hani Hafez

Saudi Arabia

Linda Halcon

USA

Nagaraja Haleagrahara

Australia

Helen Hall

Australia

Kathryn Hall

USA

Heidemarie Haller

Germany

Sherifa Hamed

Saudi Arabia

Ann Hamilton

USA

Chris Hamilton

United Kingdom

Katherine Hammer

Australia

Maryam Hamzeloo Moghadam

Iran

Gajin Han

South Korea

Jma Hannan

Bangladesh

Sheng-Po Hao

Taiwan

Hideaki Hara

Japan

John Harrigan

USA

Alan Harvey

United Kingdom

Md. Mohidul Hasan

Bangladesh

Tarique Hasan

Saudi Arabia

Parisa Hasanein

Iran

Tahereh Hasanloo

Iran

Y. Hashimoto

Japan

Lahcen Hassani

Morocco

Kenji Hata

Japan

Cheryl Hawk

USA

Maria Hayes

Ireland

Banasri Hazra

India

Jun He

China

Xiaojuan He

China

Ulla Hedegaard

Denmark

Horacio Heinzen

Uruguay

Elke Heiss

Austria

Andrzej Hendrich

Poland

Brian Hinote

USA

Tobias Hirsch

Germany

Kathleen Hoeger

USA

Stefan Hofmann

USA

Johanna Hok

Sweden

Carla Holandino

Brazil

Zhenfeng Hong

China

Mariana Hort

Brazil

Seyed Jalal Hosseinimehr

Iran

Mohammad Javad Hosseinzadeh

Iran

Katarina Hostanska

Switzerland

Lin Hou

China

Siobhan Howard

Ireland

Hsin Hsiu

Taiwan

Dan-Ning Hu

USA

Shengshou Hu

China

Yuanjie Hu

USA

A-Min Huang

Taiwan

Guan-Jhong Huang

Taiwan

Tur-Fu Huang

Taiwan

Xuedan Huang

Japan

Yi Huang

USA

Yu Huang

China

Jeongeun Huh

South Korea

Claire Hulme

United Kingdom

Yun Ying Hung

Taiwan

Kent Hunter

USA

Ryan Hunter

USA

Douglas Hurst

USA

Abdullah Ijaz Hussain

Pakistan

Jamal Hussaini

Malaysia

Minwoo Hwang

South Korea

Shinn-Jang Hwang

Taiwan

Savarimuthu Ignacimuthu

India

Kinichi Imura

Japan

Sinan Ince

Turkey

Masatoshi Inden

Japan

Charles Isaacs

USA

Liz Isenring

Australia

Yoichiro Isohama

Japan

P.J. Wills

India

Muazzam Jacobs

South Africa

Samineh Jafari

Iran

Saravana Kumar Jaganathan

India

Morana Jaganjac

Qatar

Ganesh Chandra Jagetia

India

Sediqeh Jalali

Iran

Naveena Janakiram

USA

Arshad Javaid

Pakistan

Ananthasankaran Jayalekshmy

India

Beom S. Jeon

South Korea

Jae-Gyu Jeon

South Korea

Vivekanand Jha

India

William Jia

Canada

Hongchi Jiang

China

Yueming Jiang

China

Yuyang Jiang

China

Liu Jianxun

China

Yongri Jin

USA

Jadwiga Jodynis-Liebert

Poland

Paul Jones

United Kingdom

Miek Jong

Netherlands

Myungsoo Joo

South Korea

Seong Soo Joo

South Korea

Amita Joshi

India

Angela Jozala

Brazil

Zhao Jun

China

Helgi Jung

Mexico

Prasad K V S R G

India

Miroslava Kačániová

Slovakia

Yearul Kabir

Bangladesh

Agnieszka Kaczor

Poland

Martina Kadmon

Germany

Cigdem Kahraman

Turkey

Léocadie Kamagaju

Belgium

Guy Paulin Kamatou

South Africa

Tetsuro Kamiya

Japan

Niranjan Kanaki

India

Kazuki Kanazawa

Japan

Masayuki Kaneko

Japan

Wenyi Kang

China

Shung-Te Kao

Taiwan

Abidemi Kappo

South Africa

Thirunethiran Karpagam

India

Devarajan Karunagaran

India

Violet Kasabri

Jordan

Naoki Kashihara

Japan

Krzysztof Kassolik

Poland

Ichiro Katayama

Japan

David Katerere

South Africa

Gurinder Kaur

India

Srini Kaveri

France

Kenji Kawakita

Japan

Hibiki Kawamata

USA

Sakineh Kazemi Noureini

Iran

K.B. Harikumar

India

William Kelly

New Zealand

Paula Kersten

United Kingdom

Young-Sam Keum

South Korea

Raghu Kg

India

Arif-Ullah Khan

Pakistan

Feroz Khan

India

Gazala Khan

USA

Haider Khan

India

M.Badruzzaman Khan

USA

Muhammad Khan

Pakistan

Rehan Khan

Canada

Shafaat Khan

Pakistan

Mahmoud Khattab

Egypt

Mohamed T. Khayyal

Egypt

Nafiseh Khosravi

Iran

Sung Hwan Ki

South Korea

Gunver Kienle

Germany

Sharon Kilbreath

Australia

Ae Ra Kim

USA

Hee Young Kim

South Korea

Hong-Hee Kim

South Korea

Hyun Jik Kim

South Korea

Hyung-Min Kim

South Korea

Jong Bin Kim

South Korea

Jong-Choon Kim

South Korea

Mi-Yeon Kim

South Korea

Seong Yun Kim

South Korea

Seung-Tae Kim

South Korea

Song-Yi Kim

South Korea

Sung Kim

South Korea

Hollis King

USA

Muge Kiray

Turkey

Emma Kirby

Australia

Roman Kireev

Spain

Victor Kiri

United Kingdom

Taro Kishida

Japan

Anoop Kishore

India

Anette Kjellgren

Sweden

Petra Klose

Germany

Ken Kobayashi

Japan

Kimberly Koester

USA

Byunghee Koh

South Korea

Eiji Kohmura

Japan

Pranawa Koirala

Nepal

Akiko Kojima-Yuasa

Japan

Ladislav Kokoska

Czech Republic

Aydin Köksal

Turkey

Ufuk Kolak

Turkey

Sevgi Kolayli

Turkey

Badireenath Konkimalla

India

Muhsin Konuk

Turkey

Imhoi Koo

USA

Sungtae Koo

South Korea

Radava Korac

Serbia

Demetrios Kouretas

Greece

Stanisław Kowalski

Poland

Nandakumar Krishnadas

India

Soundararajan Krishnaswamy

Saudi Arabia

Keitaro Kubo

Japan

Tuba Küçükkilinç

Turkey

Jules-Roger Kuiate

Cameroon

Anil Kumar

India

Dinesh Kumar

India

Nitesh Kumar

India

Pravin Kumar

India

Sanath Kumar

USA

Suresh Kumar

India

Vijay Kumar

India

Vijaya Kumar

India

Vikas Kumar

India

Prasanna Kumar Sp

India

Tatjana Kundakovic

Serbia

Joydeb Kumar Kundu

Bangladesh

Vijayanarayana Kunhikatta

India

Ramkumar Kunka Mohanram

India

Amit Kunwar

India

Hann-Chorng Kuo

Taiwan

Sajeera Kupittayanant

Thailand

Gino Kurian

India

Masahiko Kurokawa

Japan

Dong-Yeul Kwon

South Korea

Hokeun Kwon

USA

Laura López

Argentina

Daniel Lacorazza

USA

Elena Ladas

USA

Joao Lago

Brazil

Jung Nien Lai

Taiwan

Helene Langevin

USA

Thomas Langmann

Germany

George Lansner

USA

Malinee Laopaiboon

Thailand

Chantal Lau

USA

Romy Lauche

Germany

Dana Lawrence

USA

Olga Leaños

Mexico

Bombi Lee

South Korea

Bong Hyo Lee

South Korea

Choong-Gu Lee

South Korea

Eui Ju Lee

South Korea

Hyunsook Lee

South Korea

Junhee Lee

South Korea

Myeong Soo Lee

South Korea

Myung Koo Lee

South Korea

Sang Yeol Lee

South Korea

Sanghun Lee

South Korea

Yi-Jang Lee

Taiwan

Antonella Leone

Italy

Kristina Leuner

Germany

Albert Leung

Hong Kong

Lai Leung

Hong Kong

Ping-Chung Leung

Hong Kong

Arkene Levy

Jamaica

George Lewith

United Kingdom

Feng Li

USA

Fuzhong Li

USA

Hecheng Li

China

Jiansheng Li

China

Min Li

USA

Ping Li

China

Shao Li

China

Tsai-Chung Li

Taiwan

Xican Li

China

Xiu-Min Li

USA

Zhiyong Li

China

Carol Liang

USA

Fan-Rong Liang

China

Ben-Yang Liao

Taiwan

Liunn-Wang Liao

Taiwan

John Licciardone

USA

Byungmook Lim

South Korea

Micheline Lima

Brazil

Christine Limbers

USA

Thawornchai Limjindaporn

Thailand

Diingbo Lin

USA

Li-Chan Lin

Taiwan

Rong Lin

China

Sam Lin

New Zealand

Yun-Lian Lin

Taiwan

Klaus Linde

Germany

Shoei-Yn Lin-Shiau

Taiwan

Ingrid Liodden

Norway

Hans-Peter Lipp

Switzerland

Simona Carmen Litescu

Romania

Chenghai Liu

China

Dongbo Liu

China

Jer-Yuh Liu

Taiwan

Jianping Liu

China

Jiaren Liu

USA

Min Liu

USA

Quanhong Liu

China

Shing-Hwa Liu

Taiwan

Sing Kai Lo

Hong Kong

Carl Lombard

South Africa

Andrew Long

United Kingdom

Chunlin Long

China

Robert Longmore

Australia

Norberto Lopes

Brazil

Meei-Fang Lou

Taiwan

Princy Louis Palatty

India

Aiping Lu

Hong Kong

Bin Lu

China

Jin-Jian Lu

Macao

Ko-Hsiu Lu

Taiwan

Xiao Mei Lu

China

Zhigang Lu

China

John Ludbrook

Australia

Iréne Lund

Sweden

Lg Luo

USA

Bhanda Ma

India

Jin Yeul Ma

South Korea

Ruth Macdonald

USA

Mohammad Mahreghi

Iran

Cristiane Maia

Brazil

Hideyuki J. Majima

Japan

Runner R. T. Majinda

Botswana

Juraj Majtan

Slovakia

Avijit Majumdar

USA

Nasrullah Malik

Pakistan

Surulivelrajan Mallayasamy

India

Azadeh Manayi

Iran

Chitra Mandal

India

Santi M Mandal

India

Shyamapada Mandal

India

Prasenjit Manna

India

Fazliana Mansor

Malaysia

Thankamani Marar

India

Wolfgang Marktl

Austria

Manuel Martinez Garcia

Spain

Ramon Martinez-Pacheco

Spain

Giovanna Masala

Italy

Amos Massele

Botswana

Dieudonné Massoma Lembè

Cameroon

Yuki Masuda

Japan

José Matés

Spain

Jessy Mathew

India

Ivana Matic

Serbia

Adam Matkowski

Poland

Thabe Matsebatlela

South Africa

Yutaka Matsuda

Japan

Isao Matsui-Yuasa

Japan

Brian Matthews

Australia

Ney Mattoso

Brazil

Ahmet Mavi

Turkey

Musalmah Mazlan

Malaysia

Jean-Xavier Mazoit

France

Malcolm Mccoubrie

United Kingdom

Alison Mcdermott

USA

Lyndy Mcgaw

South Africa

Manoj Medal

India

Ahmed Mediani

Algeria

Omarnoel Medina

Mexico

V. Medina Villaamil

Spain

Richard Meenan

USA

Ashish Mehta

Singapore

Darshan Mehta

USA

Rajendra Mehta

USA

Martina Meinke

Germany

Eleni Melliou

Greece

Fan-Gang Meng

China

Linghua Meng

China

Lahouel Mesbah

Algeria

Marta Mesias

Spain

Yutaka Mifune

Japan

Jeremy Miles

USA

Gardenia Militão

Brazil

Maria Leticia Miranda Fernandes Estevinho

Portugal

Ashwini Mishra

USA

K.P. Mishra

India

Aparna Misra

India

Sylvia Mitchell

Jamaica

Megha Mittal

India

Tempei Miyaji

Japan

Yoko Miyasaki

USA

Kazuo Miyashita

Japan

Yasuyoshi Miyata

Japan

Unnikrishnan Mk

India

Ravishankara Mn

India

Ali Mobasheri

United Kingdom

Md. Mizanur Rahman Moghal

Bangladesh

Saleh Mohamed

Saudi Arabia

Suhaila Mohamed

Malaysia

Tarek Mohamed

Egypt

Abdul Khader Mohammed

Saudi Arabia

Maradumane Mohan

USA

Yasmin Mohd Yusof

Malaysia

Thomas Mohr

Austria

Phatlane William Mokwala

South Africa

Alex Molassiotis

Hong Kong

Fernando P. Molina-Heredia

Spain

Gloria María Molina-Salinas

Mexico

Abu-Asab Mones

USA

Robson Monteiro

Brazil

Valerio Monteiro-Neto

Brazil

Changjong Moon

South Korea

Sung-Kwon Moon

South Korea

Glauco Morales

Chile

Tatsuya Moriyama

Japan

Mohamed Morsy

Egypt

Said Moselhy

Saudi Arabia

Mainen Moshi

Tanzania

Mahbub Mostofa

Bangladesh

Ramzi Mothana

Saudi Arabia

Shan Mou

China

Kamal Moudgil

USA

Laila Moujir

Spain

Mack Moyo

South Africa

Jayesh Mudgal

India

Aliyu Muhammad

Nigeria

Naveed Muhammad

Pakistan

Alek Mukerjee

India

Carla Carolina Munari

Brazil

William Mundy

USA

Renuka Munshi

India

Theresa Munyombwe

United Kingdom

Akira Murakami

Japan

Dudzai Mureyi

Zimbabwe

Mark Musch

USA

Novatus Mushi

Tanzania

Mohd Rais Mustafa

Malaysia

Raghuram Nagarathna

India

Richard Nahin

USA

Ashalatha Nair

India

Toshiya Nakamura

Japan

Lia Nakao

Brazil

Madhavan Nampoothiri

India

Nima D Namsa

India

Chanin Nantasenamat

Thailand

Edmund Nartey

Ghana

Christina Nasadyuk

Ukraine

José Nascimento

Brazil

Nizar Nasri

Tunisia

Urs Nater

Switzerland

Moises Navarro

Mexico

Yogendra Nayak

India

Jolanta Nazaruk

Poland

Ernest Ng

Hong Kong

Simon Ng

Hong Kong

Tzi Bun Ng

Hong Kong

Long Nguyen

Singapore

Endalkachew Nibret

Ethiopia

Glenda Nicioli Da Silva

Brazil

Janne Nielsen

Denmark

Nameirakpam Nirjanta Devi

India

Frederic Nico Njayou

Cameroon

Cristina Nogueira

Brazil

Hedvig Nordeng

Norway

Silvana Noviello

Italy

Akito Nozaki

Japan

Yoshizu Nozawa

Japan

Mohd Fahami Nur Azlina

Malaysia

Emeka Nweze

Nigeria

Henrietta Ayodele Oboh

Nigeria

Kylie O'Brien

Australia

Oluwafemi Oguntibeju

South Africa

Mikako Oka

Japan

Yoshihiro Okamoto

Japan

Festus Okoye

Nigeria

Simone Oliveira

Brazil

Doreen Oneschuk

Canada

Nada Orsolic

Croatia

Pauline Osamor

Nigeria

Mike Osei-Atweneboana

Ghana

Thomas Ostermann

Germany

Nor Hayati Othman

Malaysia

Romy Ouziel

Belgium

Vincent Brice Owona Ayissi

Cameroon

Keriman Ozadali

Turkey

Alessio Paffoni

Italy

Andreas Pahl

Germany

Aravinda Pai

India

Sokcheon Pak

Australia

Dhanabal Palanisamy

India

Shanthi Palanivelu

India

Jesús Palá-Paúl

Spain

Jerzy Palka

Poland

Min-Hsiung Pan

Taiwan

Siyi Pan

China

Abhay K. Pandey

India

Akhilesh Pandey

USA

Badri N. Pandey

USA

Virendra Pandey

USA

Nancy Pandita

India

Uraiwan Panich

Thailand

Hi-Joon Park

South Korea

Jae-Woo Park

South Korea

Kye Won Park

South Korea

Sang-Joon Park

South Korea

Seong-Uk Park

South Korea

Wansu Park

South Korea

Yong-Ki Park

South Korea

Seema Patel

India

Charlotte Paterson

United Kingdom

Anum Pathan

Pakistan

Santosh Patil

India

Pimpicha Patmasiriwat

Thailand

Sittiporn Pattaradilokrat

Thailand

Niraldo Paulino

Brazil

Jun Peng

China

Wen-Huang Peng

Taiwan

Andrés Pereañez

Colombia

Ermelinda Pereira

Portugal

Patricia Pereira

Portugal

Jenny Persson

Sweden

Vera Lucia Petricevich

Mexico

Florian Pfab

Germany

Preecha Phuwapraisirisan

Thailand

Jaqueline Picada

Brazil

Stefano Pieretti

Italy

Andrzej Pilc

Poland

Karen Pilkington

United Kingdom

Frederico Pittella

Japan

Boris Pliyev

Russian Federation

Luis Alberto Ponce-Soto

Brazil

Ruxandra Popescu

Austria

Ada Popolo

Italy

Werayut Pothitirat

Thailand

Jalal Pourahmad

Iran

Kirti Prabhu

India

Gabriel-Ioan Prada

Romania

Komal Prasad

India

Gbks Prasadf

India

K Prashanth

India

John Preshanth Kumar

India

Gerhard Prinsloo

South Africa

Sonya Pritzker

USA

Shuhong Qiao

USA

Ji-Shu Quan

China

Cassandra Quave

USA

Ana Quesada

Spain

Jael Quintero

Mexico

Rahmatullah Qureshi

Pakistan

Shamim Qureshi

Pakistan

Nisaudah Radenahmad

Thailand

Mania Radfar

United Kingdom

Mahmoud Rafieian-Kopaei

Iran

Padma Raghunathan

India

Asmah Rahmat

Malaysia

Mahendra Rai

India

Nagaraja Ts Raja

India

Sudhakar Raja

India

Raju Rajala

USA

Revathi Rajappan

India

Jayadev Raju

Canada

Gamal Ramadan

Egypt

Kota Ramana

USA

Kanchugarakoppal S Rangappa

India

Raghavendra Rao

India

Shaival Kamalaksha Rao

India

Quintin Rascon-Cruz

Federated States of Micronesia

Huma Rasheed

Pakistan

Umbreen Rashid

Pakistan

Luca Rastrelli

Italy

Azhar Rasul

China

Edward Ratovitski

USA

Shabnam Rauf

Pakistan

Shumaila Rauf

Pakistan

Jo-Anne Rayner

Australia

Daniel Redwood

USA

Vivienne Reeve

Australia

K. Dean Reeves

USA

Katie Reindl

USA

Scott Reisman

USA

Guixing Ren

China

Wanda Reygaert

USA

Chae-Seo Rhee

South Korea

Man Hee Rhee

South Korea

Daniela Rigano

Italy

Marit By Rise

Norway

Christoph Ritter

Germany

Jose Rivera

USA

Robert Zweigerdt

Germany

Tara Roberts

Australia

Paula Robson

Canada

Joseph Roscoe

USA

Kathryn Rose

Australia

Bjorn Rosengren

Sweden

Zyed Rouis

Turkmenistan

Aditi Roy

India

Bishnupada Roy

India

Ana Lúcia T G Ruiz

Brazil

Eduardo Ruiz-Bustos

Mexico

Vivian M. Rumjanek

Brazil

Victor P.M.G. Rutten

Netherlands

Olli Ruuskanen

Finland

Jong Hoon Ryu

South Korea

Leandro S. Moreira Dill

Brazil

Muhammad Saeed

Pakistan

Soodabeh Saeidnia

Iran

Zehra Safi Oz

Turkey

Sunil Sagar

Saudi Arabia

Subhrajit Saha

USA

Sumaira Sahreen

Pakistan

Cinu Sajan

India

Nasrin Samadi

Iran

Ravindra Samarth

India

Christelle Sanchez

Belgium

Enrica Santarcangelo

Italy

Serpil Sarıözkan

Turkey

Mehmet Ali Saracli

Turkey

Parisa Sarkhail

Iran

Jerome Sarris

Australia

Sreenivasan Sasidharan

Malaysia

Hiromi Sato

Japan

Johannes Saukel

Austria

Michael Schmidt

USA

Laura Schnackenberg

USA

Paul Schnitzler

Germany

Zev Schuman-Olivier

USA

Wolfgang Schwarz

Germany

Dana Seidlova-Wuttke

Germany

Shamala Devi Sekaran

Malaysia

Takashi Seki

Japan

Ruchi Badoni Semwal

South Africa

Subhabrata Sengupta

India

Je Kyung Seong

South Korea

Gautam Sethi

Singapore

Rabiaa Manel Sghaier

Tunisia

Ali Shafaghat

Iran

Nermeen Shaffie

Egypt

Gaurang Shah

India

Naseer Ali Shah

Pakistan

Ahmad Reza Shahverdi

Iran

Jeremia Shai

South Africa

Leshweni Jeremia Shai

South Africa

Baoci Shan

China

Narkunaraja Shanmugam

India

Aranganathan Shanmuganathan

USA

Ajay Sharma

South Korea

Alok Sharma

USA

Ashoke Sharon

India

Wei Shen

USA

Xueyong Shen

China

Rekha Shenoy

India

Gajanan Sherbet

United Kingdom

Dazhuo Shi

China

Jingshan Shi

China

Feng-Shiun Shie

Taiwan

Yuan-Wei Shih

Taiwan

Shigeomi Shimizu

Japan

Hiroshi Shimoda

Japan

Byung-Cheul Shin

South Korea

Brian Shiple

USA

Mohammad Shoeb

USA

Yun Hee Shon

South Korea

Rupal Shrivastava

India

Ahmad Nazrun Shuid

Malaysia

Erica Sibinga

USA

Bina Siddiqui

Pakistan

pmes Sil

India

Damaris Silveira

Brazil

Rakesh Sindhu

India

Zia-Ud-Din Sindhu

Pakistan

Inderpal Singh

USA

Satendra Singh

South Africa

Raghu Sinha

USA

Hande Sipahi

Turkey

Fuschia M. Sirois

Canada

Cesare Sirtori

Italy

Dan Sliva

USA

Caroline A Smith

Australia

Felix Soares

Brazil

Eun-Hwa Sohn

South Korea

Youngjoo Sohn

South Korea

Didem Sohretoglu

Turkey

Mohamed Soliman

Saudi Arabia

Maha Soltan

Egypt

Chang Gue Son

South Korea

Hwa-Young Son

South Korea

Chi Young Song

USA

Marcos Soto-Hernandez

Mexico

Carla Sousa

Portugal

Maria João Sousa

Portugal

Abimbola Sowemimo

Nigeria

Sarangapani Sreelatha

Singapore

Bungorn Sripanidkulchai

Thailand

Anshu Srivastava

India

Andrew Stachulski

United Kingdom

Anthony Staines

Ireland

Agata Stanek

Poland

Tatjana Stanojkovic

Serbia

Elena Stashenko

Colombia

Grzegorz Stasilojc

Poland

Amie Steel

Australia

Vanessa Steenkamp

South Africa

Christopher Stevenson

Australia

Joanna Stewart

New Zealand

Gordana Stojanovic

Serbia

Kent Stuber

Canada

Britt Stuge

Norway

Barbara Stussman

USA

Chin-Cheng Su

Taiwan

Chun-Li Su

Taiwan

Wei-Juin Su

Taiwan

Alírica Suárez

Venezuela

Prasanna Subbarao

India

Sorimuthu Subramanian

India

Ganapasam Sudhandiran

India

Lonchin Suguna

India

Kevin Sun

New Zealand

Nak Ju Sung

South Korea

Taofik Sunmonu

South Africa

Sekhar Surapaneni

USA

S V Suresh Kumar

India

Martina Sutovska

Slovakia

Yukiya Suzuki

Japan

Naveed Ahmed Syed

India

Yt Szeto

Hong Kong

T.A. Ajith

India

Helen Taggart

USA

Kae Takagi

Japan

Reinaldo Takahashi

Brazil

Kazuhiro Takemoto

Japan

Siavash Talebi Taher

Denmark

Bender Tamas

Hungary

Jean De Dieu Tamokou

Cameroon

Chanderdeep Tandon

India

Chih-Hsin Tang

Taiwan

Giovanni Tarantino

Italy

Tomasz Tarko

Poland

Anil Tatiya

India

Ashraf Taye

Egypt

Gerald Ngo Teke

Cameroon

Nitin Telang

USA

Krishnaraj Thirugnanasambantham

India

Menaka Thounaojam

India

Anne Tiedemann

Australia

Jon Tilburt

USA

Stephanie Tjen-A-Looi

USA

Prasad Tollamadugu

India

Christy Tomkins-Lane

Canada

Li Tong

China

Fátima Torrico

Venezuela

Marcio Torsoni

Brazil

Jaime Tortoriello

Mexico

Karine Toupin April

Canada

Tuğba Toz

Turkey

Danielle Trentin

Brazil

Jesus Tresguerres

Spain

Edvaldo Trindade

Brazil

Pankaj Tripathi

India

Shalini Tripathi

India

Debu Tripathy

USA

Ritu Trivedi

India

Chin-Chuan Tsai

Taiwan

Hector W.H. Tsang

Hong Kong

William Tsang

China

Fumio Tsuji

Japan

Takahiro Tsujita

Japan

Koki Tsuruoka

Japan

Nattanan T-Thienprasert

Thailand

Tania Ueda-Nakamura

Brazil

Mariko Uehara

Japan

Rizwan Ul Haq

Pakistan

Sigurd Uldall

Denmark

Gerhild Ullmann

USA

Sandra Ulrich-Rückert

Germany

Solomon Umukoro

Nigeria

Mehmet Ünlü

Turkey

Oya Unsal Tan

Turkey

Kumud Upadhaya

India

Dawn Upchurch

USA

Ozge Uysal Soyer

Turkey

Hitesh Vaidya

Canada

Padmaja Vaidyanathan

India

Dora Valencia

Mexico

Margaret Diana Van Die

Australia

Leo Van Griensven

Netherlands

Sandy Van Vuuren

South Africa

Robyn Van Zyl

South Africa

Ina Vandebroek

USA

Clarisse Vandebrouck

France

Luca Vanella

Italy

Emmelyne Vasse

Netherlands

Mani Vasudevan

India

Mahdi Vazirian

Iran

Carlos Velazquez

Mexico

Gopalakrishnan Velliyur Kanniappan

India

Balaiya Velmurugan

USA

Thirumurthy Velpandian

India

Ramaprabhu Vempati

India

Melissa Vetten

South Africa

Maria Do Carmo Vieira

Brazil

Kuruba Vijaya Bhaskar

India

Patricia Vit

Venezuela

Annabella Vitalone

Italy

Luis Vitetta

Australia

Jarupa Viyoch

Thailand

Gustavo Volpato

Brazil

Jiri Vrba

Czech Republic

Ishrat Waheed

Pakistan

Shinya Wakusawa

Japan

Marianne Wallis

Australia

Charles Wambebe

Uganda

Chong-Zhi Wang

USA

Cj Wang

China

Guangjun Wang

China

Haiyan Wang

China

Hui-Min Wang

Taiwan

Jia-Bo Wang

China

Jianwei Wang

China

Junpeng Wang

USA

Peigang Wang

Hong Kong

Ronald Wang

Hong Kong

Shengbiao Wang

USA

Xiao Wang

China

Xiaoying Wang

USA

Yingwei Wang

China

Yongli Wang

China

Zilai Wang

USA

Jon Wardle

Australia

Jintanaporn Wattanathorn

Thailand

Peter Wayne

USA

Helen Weatherly

United Kingdom

Xin Wei

USA

Michael Weingarten

Israel

Nancy Wellman

USA

Rebecca Wells

USA

Ursula Werneke

Sweden

Peter White

United Kingdom

Jenny Wilkinson

Australia

Karen Willis

Australia

Stephanie Willson

USA

Michael Wink

Germany

Wipawee Winuthayanon

USA

Yuvadee Wongkrajang

Thailand

Samyong Woo

South Korea

Lisa Wood

USA

Julia Woodman

United Kingdom

Randy Worobo

USA

Chi-Rei Wu

Taiwan

Darong Wu

China

Geng Wu

China

Ming-Jiuan Wu

Taiwan

Nan Wu

China

Xiaoke Wu

China

Mansuang Wuthi-Udomlert

Thailand

Lesley Wye

United Kingdom

Leder Xavier

Brazil

Helen Xiao

China

Jianping Xie

China

Jing Xie

China

Donghui Xu

China

Jinjin Xu

China

Jinsen Xu

China

Ke Xu

China

Lin Xu

Hong Kong

Qianqian Xu

China

Yun Xu

China

Zhi-Xiang Xu

USA

Arun Yadav

India

Nobuo Yamaguchi

Japan

Yoshihisa Yamashita

Japan

Chunhong Yan

USA

Bin Yang

United Kingdom

Chang-Hai Yang

China

Chia-Ron Yang

Taiwan

Jian Yang

Canada

Junjun Yang

Hong Kong

Peiying Yang

USA

Shun-Fa Yang

Taiwan

Sien-Hung Yang

Taiwan

Wen-Chin Yang

Taiwan

Woong Mo Yang

South Korea

Xiu-Wei Yang

China

Yulin Yang

Taiwan

Zhang Yanjun

China

Akira Yano

Japan

Pei-Li Yao

USA

Wei Yao

China

Zhong-Xiang Yao

China

Eric Yarnell

USA

Ching-Hua Yeh

Taiwan

Gloria Yeh

USA

Jwu-Lai Yeh

Taiwan

Chia Jui Yen

Taiwan

Mijung Yeom

South Korea

Wing-Fai Yeung

Hong Kong

Chenjiang Ying

Japan

Zafer Yonden

Turkey

Hwan-Soo Yoo

South Korea

Seung-Schik Yoo

USA

Jarred Younger

USA

Susana Zacchino

Argentina

Fatheya Zahran

Saudi Arabia

Hilal Zaid

Israel

Galia Zamaratskaia

Sweden

Stella Zamuner

Brazil

Keivan Zandi

Malaysia

Anand Zanwar

India

Mirjam Zeisel

France

Anthony Zhang

Australia

Boli Zhang

China

Chunling Zhang

China

Glenn Zhang

USA

Hua Zhang

China

Paul Zhang

USA

Qiqing Zhang

China

Tong Zhang

China

Xiaowei Zhang

USA

Yan Zhang

USA

Yunguang Zhang

China

Zunjian Zhang

China

Jun Zhao

China

Limin Zhao

China

Ling Zhao

China

Ming-Gao Zhao

China

Xiaohu Zhao

China

Xiaoshan Zhao

China

Yonghua Zhao

Macao

Yunxue Zhao

China

Zhen Zheng

Australia

Jian-Jiang Zhong

China

Yifei Zhong

China

Jin-Rong Zhou

USA

Kehua Zhou

USA

Kequan Zhou

USA

Wei Zhou

USA

Bing Zhu

China

Xuan Zhu

China

Zhitu Zhu

China

Suzanna Zick

USA

Karine Rigon Zimmer

Brazil

Denis Zofou

Cameroon

Ping Zou

Japan

Istvan Zupko

Hungary

## Pre-publication history

The pre-publication history for this paper can be accessed here:

http://www.biomedcentral.com/1472-6882/14/13/prepub

